# SMURF2 in Anticancer Therapy: Dual Role in Carcinogenesis and Theranostics

**DOI:** 10.3390/ijms27031538

**Published:** 2026-02-04

**Authors:** Joy Eom, Yejin Chun, Hae Ryung Chang

**Affiliations:** 1 Department of Biological Sciences, Korea Advanced Institute of Science and Technology, Daejeon 34141, Republic of Korea; joyeom@kaist.ac.kr; 2 Department of Life Science, Handong Global University, Pohang 37554, Republic of Korea; yejinchun0624@gmail.com

**Keywords:** SMURF2, E3 ligase, targeted therapy, biomarker, anticancer agent, TGF-β signaling pathway

## Abstract

Cancer is a heterogeneous disease at the cellular level and analyzing the genetic and molecular profile is essential for targeted therapy. Cancer cells continue to mutate, often resulting in drug resistance. In addition, cancers such as triple-negative breast cancer (TNBC) lack the target proteins used in some of the most effective therapies. This necessitates the identification of novel target proteins and biomarkers for effective treatment strategies. Ubiquitin E3 ligases are often differentially expressed in cancer cells, and numerous anticancer agents have been developed to inhibit them. SMURF2 is an E3 ligase that is differentially expressed in multiple cancer types. Although inhibiting upregulated SMURF2 may be strategically straightforward, enhancing the downregulated gene is often difficult. In addition, because E3 ligases ubiquitinate a variety of substrate proteins, targeting SMURF2 requires detailed analysis to achieve anticancer effect. This review discusses the dual role of SMURF2 in carcinogenesis and addresses the complex context-dependent function of SMURF2 in the various cellular pathways. In addition, resistance to existing cancer therapy related to SMURF2 and sensitivity mechanisms is discussed. Lastly, theranostic strategies for anticancer agents and biomarker development are suggested.

## 1. Introduction

Among the hallmarks of cancer, the activation of growth signaling factors and positive regulation of the cell cycle are crucial mechanisms for tumor progression [1,2,3]. These signaling factors are attractive targets for therapeutic strategies, and many of them have been extensively explored in the development of targeted therapies. However, the biological function of a given gene or protein is not always uniform across different cancer types. Depending on the tumor context, certain molecules may act as oncogenic drivers in some cancers while serving as tumor suppressors in others. Therefore, recognizing and understanding the duality of the factors is critical in drug discovery and development. This functional duality has been well documented in several key molecules.

One such example is NOTCH1, which is frequently found to be overexpressed or constitutively activated, functioning as a major oncogenic driver in various malignancies, including T-cell acute lymphoblastic leukemia (T-ALL) [4], hepatocellular carcinoma (HCC) [5], and prostate cancer [6]. Multiple NOTCH1 inhibitors, including γ-secretase inhibitors (GSIs), transcriptional blockers, and monoclonal antibodies, have been developed and have entered clinical evaluation across multiple malignancies [7,8,9,10,11,12]. Notably, overexpression of NOTCH1 in T-ALL drives leukemogenesis through aberrant activation of downstream signaling pathways, and pharmacological inhibition using GSIs successfully kills T-ALL cells by targeting NOTCH1 signaling [13]. However, NOTCH1 was found to be tumor-suppressive in head and neck squamous cell carcinoma (HNSCC) [14,15], as the restoration of NOTCH1 signaling in cells markedly suppressed tumor growth in vitro and in vivo. ARID1A is another example displaying a dual role in cancer progression [16]. Sun et al. demonstrated using hepatocellular carcinoma (HCC) mouse models that Arid1a overexpression at early tumor stages accelerated initiation, whereas liver-specific *Arid1a* knockout reduced tumor burden and incidence. In contrast, *Arid1a* loss at a later stage promoted invasion and metastasis, while in the human HCC cell line, restoration of ARID1A or its downstream targets reversed these effects [17]. Clinical data also support this opposing role, depending on the stage, showing that high ARID1A expression correlates with poor prognosis in early-stage HCC patients but serves as a protective factor in advanced-stage HCC patients [18]. Collectively, these studies highlight the context-dependent dual roles of cancer-associated genes, demonstrating that their biological functions can shift between oncogenic and tumor-suppressive depending on cellular, molecular, or tissue-specific contexts.

The ubiquitin pathway has been investigated as a therapeutic target for various diseases, including cancer [19,20]. Many are overexpressed or dysregulated in cancers and thus represent promising therapeutic targets. Among these, E3 ubiquitin ligases have attracted particular attention as potential target proteins in cancer, given their essential role in regulating protein stability and signaling networks. Several inhibitors targeting E3 ligases have been developed and entered clinical evaluation. For example, MDM2 inhibitors such as RG7388 [21,22] and SKP2 inhibitors such as curcumin [23,24,25] have shown anticancer potential and are currently under preclinical or clinical investigation. Beyond these examples, however, the development of drugs directly targeting E3 ligases remains relatively limited due to the inherent complexity of E3 ligases. The major functions of these ligases include tagging substrate proteins for proteasomal degradation and mediating non-degradative ubiquitination that regulates signaling pathways by recruiting downstream interaction partners [26,27]. Each E3 ligase interacts with numerous substrates and signaling partners; therefore, its inhibition can elicit diverse biological effects depending on the cellular context [28]. For instance, RNF126, a Really Interesting New Gene (RING)-type E3 ubiquitin ligase, has emerged as a promising therapeutic target due to its oncogenic functions [29,30]. Its functional significance was first highlighted in the ubiquitination and proteolysis of mislocalized proteins [31] and then in the proteolysis of mistranslated proteins [32,33]. Its upregulation and overexpression have been reported in multiple cancers, including colorectal [34], lung [35], liver [36], gastric [37], ovarian [38], prostate [39], and pancreatic cancers [40]. Vu et al. have shown that overexpression of RNF126 enables ovarian cancer cells to evade cell death and colonize in the peritoneal fluid [38]. Using breast and prostate cancer cell lines, Zhi et al. showed that RNF126 knockdown decreased cell viability, induced G1–S arrest, and increased p21 protein levels, revealing that RNF126 promotes cell-cycle progression through p21 degradation [41]. In addition, RNF126 was shown to restore cell proliferation in bladder cancer cell lines by polyubiquitinating PTEN [42]. However, RNF126 has been reported to have tumor suppressive functions. For example, in MCF7 cells, RNF126 facilitated BRCA1 transcription by interacting with E2F1 [43]. Its depletion reduces BRCA1 expression and thus disrupts homologous recombination, increasing genomic instability. Paradoxically, such instability sensitizes cancer cells to PARP inhibitors through synthetic lethality [44]. Deeper investigation into the substrate specificity and interaction networks of individual E3 ligases is essential to elucidate their precise functions in tumorigenesis and to design therapeutic strategies that are responsive to specific cellular contexts.

Smad ubiquitin regulatory factor 2 (SMURF2), a member of the HECT-type E3 ubiquitin ligases, is considered a potential molecular target for cancer therapy and has been steadily investigated for its roles in tumor development and progression [45]. The role of SMURF2 was first identified as a negative regulator in the Transforming Growth Factor beta (TGF-β) signaling pathway [46]. The TGF-β signaling pathway is a complex network that regulates diverse cellular processes [47]. During embryogenesis, TGF-β signaling promotes cell proliferation by activating the Platelet-Derived Growth Factor beta (PDGF-β) promoter and induces Epithelial-to-Mesenchymal Transition (EMT) to regulate cell migration [48,49,50,51,52,53]. Conversely, in adult cells, TGF-β signaling suppresses proliferation by upregulating Cyclin-Dependent Kinase Inhibitors (CDKIs) [54,55], downregulating c-MYC and the inhibitor of DNA binding (ID) family [56,57,58], and inducing apoptosis [59,60,61]. Due to these diverse roles, TGF-β signaling can be either tumor-promoting or tumor-suppressive [62,63], and the deactivated developmental programs can re-surface mechanisms that induce EMT in cancer cells, resulting in invasion and metastasis [64,65,66,67,68]. On the other hand, the loss or attenuation of the tumor-suppressive function is frequently observed in early tumorigenesis, enabling uncontrolled cell growth and evasion of apoptosis [69,70,71].

SMURF2 is one of the main ubiquitin E3 ligases that regulate the stability of multiple components of the TGF-β signaling pathway [46]. In some breast cancers, SMURF2 functions as a tumor-promoting factor [72], whereas in colorectal cancer, its high expression correlates with better prognosis [73]. In addition to the TGF-β signaling pathway, SMURF2 is also involved in various essential cellular functions and pathways, such as Hedgehog signaling [74,75], DNA damage response [76,77], and cell cycle regulation pathways [78,79], and its implication in cancer is therefore complex. Given the context-dependent functions of SMURF2, systematic analysis of its cell/tissue-specific roles in diverse cancers is needed. Such an investigation could contribute to establishing new potential strategies for cancer therapy and biomarkers by targeting the SMURF2 pathway in a cancer type-specific manner. This paper highlights SMURF2’s dual tumor-promoting and tumor-suppressive role in critical cellular pathways, evaluates its relevance to current cancer therapies, and outlines novel, precise SMURF2-related cancer theragnostics.

## 2. SMURF2 and Its Function

SMURF2 is comprised of an N-terminal C2 domain, tandem WW domains that recognize PPxY motifs, and a C-terminal HECT catalytic domain; phosphorylation at Thr249 (SMURF2^Thr249^) in the inter-domain linker tunes catalytic efficiency and downstream signaling output [80,81]. It generates substrate-specific ubiquitin signals, catalyzing non-degradative mono-ubiquitination or degradative K48-linked poly-ubiquitination, modulating signaling outputs or promoting receptor turnover [82]. SMURF2 is localized both in the nucleus and the cytosol, and its localization and accessibility to specific substrates determine its downstream involvement [83].

**Figure 1:**
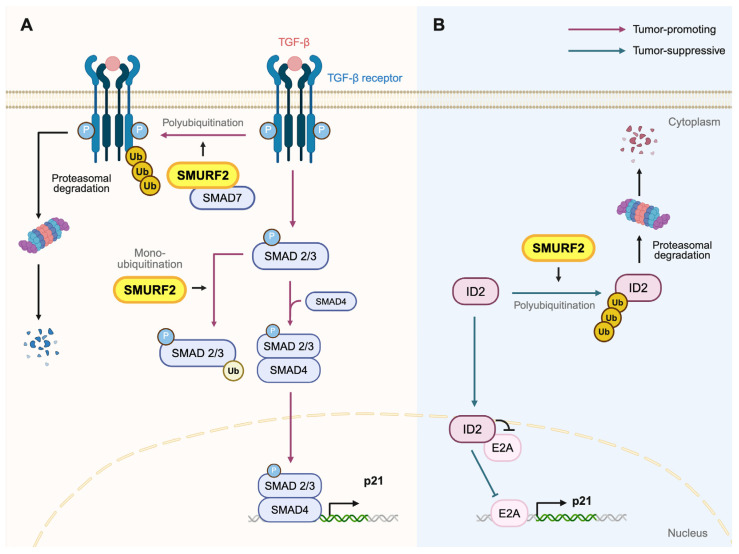
SMURF2 fine-tunes TGF-β signaling and p21 expression through dual regulation of SMAD2/3 and the ID2–E2A axis. (**A**) SMURF2 mono-ubiquitinates activated SMAD2/3, restricting their nuclear accumulation and reducing their transcriptional activity, which in turn attenuates SMAD-dependent p21 induction. In parallel, SMURF2 is recruited to the TGF-β receptor complex by SMAD7 to catalyze K48-linked polyubiquitination of the receptor, promoting its proteasomal degradation. Through coordinated regulation of both downstream SMAD effectors and receptor stability, SMURF2 fine-tunes the overall amplitude and duration of TGF-β signaling. (**B**) SMURF2 poly-ubiquitinates ID2 and targets it for proteasomal degradation, thereby relieving ID2-mediated inhibition of the transcription factor E2A. Freed E2A can then bind to the CDKN1A promoter to activate p21 transcription, supporting an independent checkpoint pathway that operates alongside canonical SMAD signaling. This SMURF2–ID2–E2A axis ensures appropriate p21 induction even when SMAD activity is restrained (created with Biorender.com, accessed on 6 January 2026).

In the cytosol, SMURF2 regulates the TGF-β pathway by controlling receptor stability and modulating the availability of downstream SMAD proteins. This pathway encompasses a variety of factors, where there are 33 genes of the TGF-β family proteins in the mammalian system [84]. In general, two cell surface receptors are required to join for downstream activation: the Type I and Type II receptors. In relation to SMURF2, TGF-β1/2/3 activation of the Type I receptor, TGF-β receptor I (TβRI or ALK5), and Type II receptor TGF-β receptor II (TβRII) is most well understood [85]. The TGF-β–TβRI–TβRII activation activates SMAD2 and SMAD3, which then interact with SMAD4. This complex is imported into the nucleus and is involved in various transcriptional activities. One of the well-known target genes is *CDKN1A* (p21), a cell cycle regulator and tumor suppressor in nature [82,86,87,88,89]. SMURF2 polyubiquitinates TβR1 through the recruitment by SMAD7, promoting receptor degradation [46]. It also polyubiquitinates multiple downstream SMAD effectors, including SMAD1, SMAD2, and SMAD3 for degradation (Figure 1A) [90]. Another mode of regulation is achieved through SMURF2-mediated mono-ubiquitination of SMAD2/3, which restricts their nuclear accumulation and transcriptional activity [82,86]. This attenuates p21 transcription, maintaining it at a level sufficient for proper cell-cycle arrest while preventing excessive or prolonged TGF-β signaling. Lastly, non-Smad signaling of the TGF-β pathway, or the noncanonical pathway, activates Ras, activating the mitogen-activated protein kinase (MAPK) ERK pathway, which is a well-established proto-oncogene [91]. This response is often delayed or occurs at a lower intensity compared to the activation via the epidermal growth factor [91,92]. Crosstalk with ERK activation is one of the reasons why TGF-β signaling can be both oncogenic and tumor suppressive. Bone Morphogenetic Protein (BMP), a TGF-β family protein, increases Ras activation and Erk1/2 in osteoblasts. Bandyopadhyay et al. have demonstrated that dermal cells activated ERK1/2, while inhibition was observed in epidermal cells [93]. This depended on TβRII expression level, which preferentially activated ERK1/2, whereas more TβRI prefer the SMAD downstream pathway. Depending on the basal level of receptors and the differentiated state of the cell type, SMRUF2 regulation of TGF-β signaling can be tumor-promoting or suppressive.

In the nucleus, SMURF2 ubiquitinates ID proteins, which are essential during development and in stem cells [58,94,95]. ID2 functions as an inhibitor of E2A by preventing its DNA-binding activity, thereby suppressing p21 transcription. In the ID2–E2A–p21 axis, SMURF2 counteracts this repression by ubiquitinating ID2 and promoting its proteasomal degradation, which frees E2A to activate p21 expression (Figure 1B) [45,96,97]. Interestingly, TGF-β is associated with ID inhibition [98], and the role of SMURF2 in this crosstalk concerning cancer will be discussed later.

**Figure 2:**
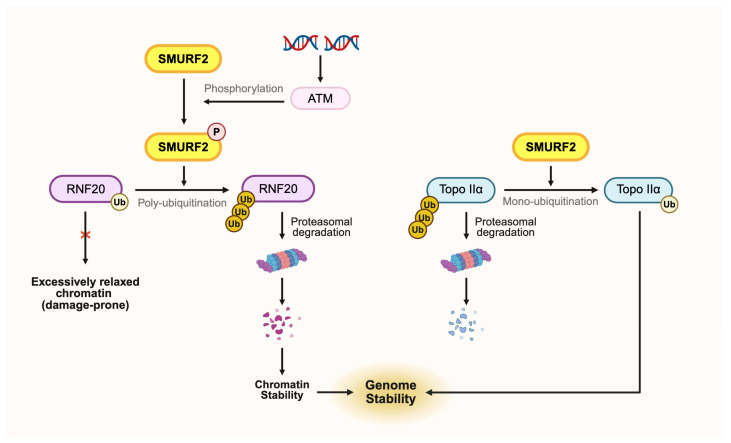
SMURF2 orchestrates genome stability through Topo IIα/ATM–RNF20 and RNF220–PRC2 regulatory axis. SMURF2 limits K48-linked polyubiquitination of Topoisomerase IIα and favors mono-ubiquitination, thereby preserving Topo IIα stability and its function in resolving DNA supercoiling. After DNA damage, ATM-activated SMURF2 ubiquitinates and downregulates RNF20 to prevent excessive H2Bub1 and restore proper chromatin compaction. Together, these actions support efficient DNA repair and maintain genome integrity (created with Biorender.com, accessed on 6 January 2026).

In the nucleus, in addition to transcription regulation, SMURF2 is a contributor to genome maintenance. It stabilizes Topoisomerase IIα (Topo IIα), which relieves DNA supercoiling stress during replication and transcription [99]. In mouse embryonic stem cell and human cancer cell lines, SMURF2 converted K48-linked polyubiquitination to mono-ubiquitination of Topo IIα. This was achieved by occupying the substrate interface to block K48-polyubiquitin chain assembly and instead attaching a stabilizing mono-Ub moiety, thereby reprogramming the ubiquitin code from a degradation signal to a stabilization outcome (Figure 2) [100,101,102]. In addition, SMURF2 regulates chromatin state through interaction with ATM and RNF20, both of which are essential for DNA double-strand break (DSB) repair. While RNF20-driven H2B mono-ubiquitination (H2Bub1) normally promotes the chromatin relaxation required for repair, excessive H2Bub1 produces an overly relaxed and structurally exposed chromatin state that is more susceptible to damage [103]. Upon DNA damage, ATM phosphorylates SMURF2, which then ubiquitinates and degrades RNF20, restoring appropriate chromatin compaction and supporting timely DNA repair (Figure 2) [76,103].

**Figure 3:**
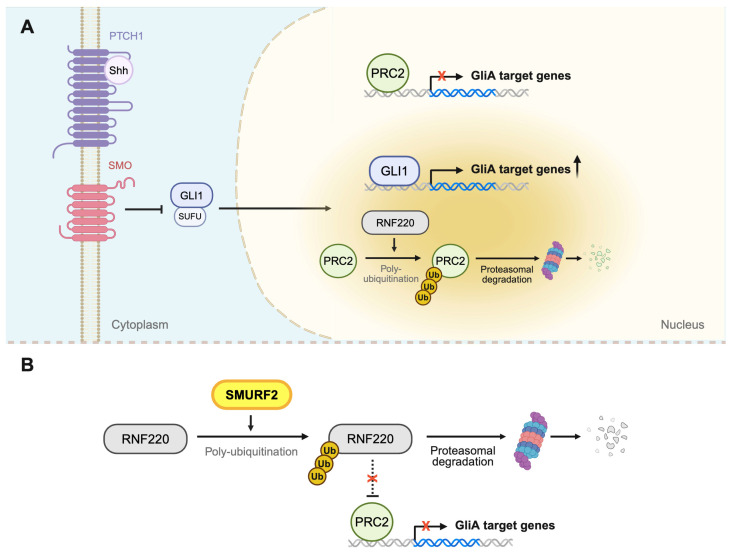
SMURF2–RNF220 axis regulates Hedgehog/GLI signaling pathway. (**A**) In the absence of SMURF2 activity, RNF220 is stabilized and promotes Hedgehog pathway activation. Under basal conditions, the PRC2 complex represses the transcription of GLI1 target genes. RNF220 ubiquitinates components of the PRC2 complex, resulting in reduced PRC2-mediated repression at GLI1 target genes and enhanced GLI1–dependent transcription. (**B**) SMURF2 mediates polyubiquitination of RNF220, leading to its proteasomal degradation. Depletion of RNF220 preserves PRC2 function at GLI1 target loci, maintaining transcriptional repression and limiting Hedgehog pathway activation (created with Biorender.com, accessed on 6 January 2026).

Other roles of SMURF2 involve modulation of the Hedgehog signaling by targeting RNF220, an E3 ligase that regulates Polycomb Repressive Complex 2 (PRC2)-mediated repression at GLI target genes [104]. Under basal conditions, the PRC2 complex maintains an H3K27me3-dependent repressive chromatin state at GLI1 target loci, thereby suppressing GLI-dependent transcription. RNF220 promotes the Hedgehog pathway by ubiquitinating components of the PRC2 complex, enhancing the expression of GLI1 target genes, including *MYC* and *SNAI1*, which are involved in cell proliferation and embryonic development (Figure 3A) [104,105]. SMURF2 mediates polyubiquitination and proteasomal degradation of RNF220, restoring the transcriptional repression at GLI1 target genes by PRC2, thereby restricting excessive Hedgehog pathway activation (Figure 3B) [104,106,107].

## 3. Dysregulation of SMURF2 in Tumorigenesis

The function of SMURF2 is regulated by its post-translational modification (PTM), which dictates the downstream effect [108,109]. Non-covalent interaction with Nedd8 via the HECT domain autoinhibits SMURF2, and Tumor Necrosis Factor Receptor-Associated Factor 4 (TRAF4) ubiquitin E3 ligase ubiquitinates the K119 residue for degradation, both enhancing the TGF-β signaling [110,111]. In skeletal development, the phosphorylation of threonine 249 by Erk5 activates SMURF2, which is important for regulating Sox9 expression by TGF-β signaling via Smad 2/3 [112]. Dysregulation of PTM factors alters the function of SMURF2 in a context-dependent manner. In addition to PTM, aberrant expression levels of SMURF2 have been observed in different types of cancer. How each condition contributes to cancer is context-driven, as diverse functions of SMURF2 have been discussed earlier. This section discusses in detail the carcinogenic effects of SMURF2-related pathways upon their dysregulation. Table 1 summarizes the tumor suppressive and oncogenic functions of SMURF2 in different cancer contexts.

### 3.1. Downregulation of SMURF2 in Tumorigenesis

TCGA dataset analysis revealed that Chromobox protein homolog 3 (CBX3) expression level is significantly increased in pancreatic cancer patients who are currently smoking [120]. In addition, cigarette carcinogens markedly increase Y-box-binding protein 1 (YBX1) expression, which elevates CBX3 transcription [120,121]. The upregulated *CBX3* represses *SMURF2* transcription, promoting tumorigenesis in pancreatic cancer cells via the TGF-β signaling pathway [120,122]. This is further compounded by TRAF4, which ubiquitinates SMURF2 for degradation through the ubiquitin–proteasome system (UPS) and simultaneously recruits Ubiquitin-Specific Peptidase 15 (USP15) to the TGF-β receptor, where it removes ubiquitin chains to prevent SMURF2-mediated receptor degradation in vitro and in vivo [111,143]. Collectively, these mechanisms shift TGF-β signaling from a homeostatic, growth-restraining program to one that supports tumor progression.

Cancer stem cells (CSCs) are often drug-resistant and show poor prognosis, and decreased levels of SMURF2^Thr249^ phosphorylation, which is the enhanced form, have been observed. Specifically, Glioma stem cells (GSCs) showed lower phosphorylation levels of SMURF2^Thr249^ than differentiated glioma cells [80,109]. In human glioblastoma (GBM) tissues, low levels of SMURF2^Thr249^ phosphorylation were observed compared to non-neoplastic brain tissue. Stabilization of the TGF-β–SMAD2/3 axis increased SOX2 level, a marker for stemness as well as tumorigenicity. Additionally, SMURF2 loss dysregulates the ID2–E2A–p21 axis, disrupting the cell-cycle control. In lung adenocarcinoma, reduced SMURF2 expression stabilizes ID2 by preventing its ubiquitin-mediated degradation. Elevated ID2 more effectively suppresses the transcription factor E2A, leading to diminished p21 expression and loss of G1 cell-cycle restraint, which in turn drives sustained proliferation and promotes tumor growth as demonstrated both in lung cancer cells and a mouse xenograft model [97].

Loss of SMURF2 also destabilizes genomic integrity by diminishing Topo IIα stability, a combination associated with elevated rates of anaphase bridges and micronuclei formation, manifestations of chromosomal instability (CIN) [100,101]. When SMURF2 is depleted, Topo IIα is prematurely degraded, resulting in defective decatenation, persistent chromosomal bridge formation, and genomic instability [100,101,102]. *SMURF2* downregulation also impairs chromatin repair capacity via the regulatory axis of SMURF2-mediated RNF20 ubiquitination and degradation. Elevation of RNF20 expression and decreased SMURF2 expression were observed in breast cancers and lymphomas [103]. As a result, excessive RNF20-driven H2Bub1 produces an overly decondensed chromatin state that increases susceptibility to DNA damage. This disrupts the coordinated recruitment of DNA repair factors, thereby delaying proper double-strand break repair [76,103]. Thus, SMURF2 downregulation compromises both chromosomal stability and chromatin repair capacity.

Another example of tumorigenesis by low SMURF2 is through its interaction with RNF220. Aberrant SMURF2–RNF220 signaling serves as a critical driver that shifts Shh pathway activity from neural progenitor maintenance to malignant growth in hedgehog-type medulloblastoma (Shh-MB). Physiologically, Shh signaling supports neural progenitor growth by enhancing GLI activator output (Figure 3). Elevated RNF220 expression can upregulate Shh signaling, driving cancer cell proliferation. Clinical Shh-MB samples demonstrated an inverse correlation between SMURF2 and RNF220 levels, and SMURF2 knockdown stabilized RNF220, increasing Shh pathway activation and promoting tumor growth in vitro and in xenograft mouse models [104,105,106,131].

Taken together, downregulation of SMURF2 dysregulates multiple axes of cellular homeostasis, TGF-β signal containment, p21 cell-cycle checkpoint, genome stability, transcriptional regulation, and the developmental pathway control, collectively shifting signaling networks toward tumor-promoting states.

### 3.2. Upregulation of SMURF2 in Tumorigenesis

In contrast to the loss-of-function, there are cases where overexpression of SMURF2 can be tumorigenic. One mechanism of expression control is achieved by microRNAs, miR-195 and miR-497. Under physiological conditions, they post-transcriptionally control SMURF2 expression to allow appropriate turnover of TβR1 and maintain balanced SMAD2/3 signaling [144]. According to the lung cancer patient tissue and blood sample database, as well as lung cancer cell line analysis, however, miR-195 and miR-497 are frequently downregulated, leading to elevated SMURF2 expression and increased ubiquitin-mediated degradation of TβR1 [144]. In in vitro and xenograft mouse models, this attenuation of TGF-β signaling reduced transcriptional induction of tumor-suppressive target p21, ultimately increasing cancer cell proliferation and tumor growth [86,90,144]. In addition, uncontrolled cell cycle progression due to SMURF2 overexpression has been associated with poor clinical outcomes across multiple cancer types [87].

Localization can also impact SMURF2 level, which is regulated by its interaction with scaffolding proteins. By analyzing human prostate and breast cancer datasets, Emanuelli et al. have identified increased levels of 14-3-3 proteins, a scaffold protein [83]. They have shown that SMURF2 undergoes relocalization by binding to cytoplasmic 14-3-3 proteins, sequestering it outside the nucleus. Unlike in normal epithelial tissues, where this shuttling is tightly regulated, in breast cancer cell lines, SMURF2 was stabilized in the cytoplasm through enhanced interaction with cytoplasmic 14-3-3 proteins, resulting in reduced nuclear import and diminished turnover [83]. As discussed earlier, SMURF2 in the nucleus is involved in chromatin stabilization, thus functions mainly as a tumor suppressor. Once stabilized in the cytoplasm, SMURF2 can switch to a growth-promoting regulator, where mitogenic pathways become increasingly dominant, changes that are directly linked to heightened proliferative capacity and increased tumor aggressiveness.

In addition, SMURF2 upregulation can over-stabilize growth signaling mediator KRAS, contributing to its oncogenic transformation. KRAS is a potent oncogene, and its expression level is regulated by a RAS family E3 ubiquitin ligase, β-TrCP1. SMURF2 monoubiquitinates its E2 ubiquitin conjugating enzyme partner, UBCH5, and forms a complex. β-TrCP1 is one of its targets for polyubiquitination, which is then processed via UPS; therefore, SMURF2 upregulation can indirectly stabilize KRAS [132,133,134,135,136]. Lung cancer patient gene expression analysis revealed a strong positive correlation between SMURF2 and KRAS expression level, and overexpression of SMURF2 stabilized KRAS in vitro [135]. Lastly, SMURF2 upregulation is involved in the tumor-promoting epidermal growth factor signaling pathway by stabilizing the receptor, EGFR. The enhanced mitogenic signaling is often associated with malignant progression of cancer and therapy resistance [87,89,137,145,146]. Ray et al. have shown that this can be achieved through the ubiquitination of EGFR by SMURF2, not for degradation, but by preventing ubiquitination by c-Cbl, another E3 ligase that ubiquitinates EGFR for degradation [137].

Through these various mechanisms, overexpression or cytoplasmic stabilization of the homeostatic tumor-suppressive SMURF2 can transform cancer cells to become more proliferative, invasive, and even treatment-resistant.

## 4. Impact of SMURF2 on Existing Therapies

Cancer cells can upregulate cellular pathways beneficial to their survival by modifying the expression of key genes, which may lead to drug resistance. SMURF2 can contribute to this process, but it can also contribute to enhancing drug sensitivity [89]. For example, the expression level of SMURF2 plays a critical role in determining cellular sensitivity or resistance to the chemotherapeutic agent, gemcitabine. Gemcitabine is an FDA-approved nucleoside analog incorporated into DNA during replication, where it terminates chain elongation and blocks DNA synthesis. It is used as standard regimens across multiple solid tumors, including pancreatic, lung, bladder, ovarian, breast, and biliary cancers [147]. However, EMT-associated resistance has been observed in pancreatic cancer, caused by the miR15b-SMURF2 axis. In pancreatic cancer patient-derived tissue samples, the SMURF2 protein level is markedly reduced and is even more decreased in gemcitabine-resistant pancreatic cancer cell lines compared to the gemcitabine-sensitive cell lines [148]. Upregulation of miR-15b degrades SMURF2, promoting TGF-β-dependent EMT, characterized by decreased E-cadherin, an epithelial marker, and increased Vimentin and Snail, which are mesenchymal markers [148]. Gemcitabine is effective in proliferative cells, and EMT can drive cancer cells into a slow-dividing, survival-focused state with low DNA-replication activity, making it gemcitabine resistant [149,150]. However, this outcome can be reversed by modulating SMURF2 levels. Inhibition of miR-15b or restoration of SMURF2 suppressed TGF-β signaling and downregulated EMT-inducing transcription factors, thereby reversing cells toward a more epithelial state, an effect that may enhance gemcitabine sensitivity in vitro [148,151]. These findings position SMURF2 as a tumor-suppressive factor that safeguards the efficacy of gemcitabine chemotherapy by restraining EMT and maintaining drug responsiveness.

**Figure 4:**
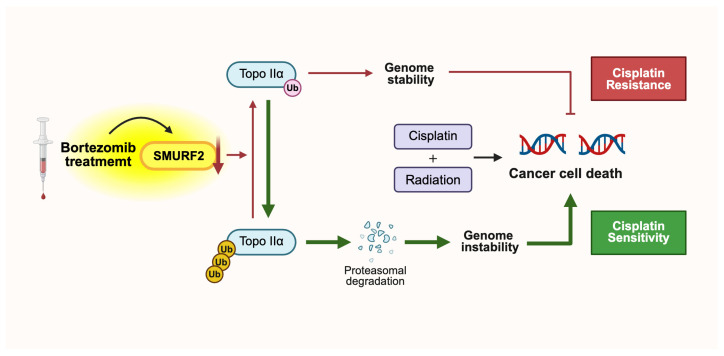
Involvement of SMURF2 in the development of resistance to cisplatin and radiation therapy. Cisplatin and Radiation-mediated severe DSB-induced apoptosis may be interrupted by the increased genome stability via SMURF2-mediated Topo IIα stabilization (created with Biorender.com, accessed on 6 January 2026).

In contrast, SMURF2 has been identified as a potential contributor to cisplatin resistance [89]. Cisplatin is a cancer chemotherapy drug that induces DNA damage-mediated apoptosis by the formation of a covalent bond between the platinum atom and guanine/adenine [152]. It was first approved by the FDA in 1978 for testicular, ovarian, and bladder cancers, later for non-small-cell lung cancer (NSCLC), and became a foundational component of standard treatment regimens for numerous additional solid tumors [153,154]. Accordingly, cisplatin resistance has emerged as a critical therapeutic challenge in NSCLC, and recent evidence indicates that targeting SMURF2 with bortezomib may represent a key strategy to overcome this resistance. Considering the cisplatin mechanism of action and the function of SMURF2 in genome maintenance (Figure 2), it can be inferred that SMURF2-mediated Topo IIα stabilization, necessary for genome maintenance, may induce cisplatin resistance by alleviating genome instability due to the cisplatin-mediated DNA damage (Figure 4). In this context, inhibiting SMURF2 activity may restore cisplatin sensitivity by enhancing cisplatin-induced DNA double-strand breaks, disrupting G2/M checkpoint control, and promoting apoptosis [89]. Bortezomib, a small-molecule proteasome inhibitor that is clinically studied and approved for various cancer treatments, can suppress SMURF2 expression [155,156], especially in human NSCLC cell lines such as PC9 and A549 [89]. Moreover, combining bortezomib with cisplatin and radiation further increased γ-H2AX-marked DNA damage and apoptosis in NSCLC cells, demonstrating a synergistic sensitizing effect. These findings, together with the observed upregulation of SMURF2 in tumors after chemoradiotherapy, support SMURF2 as a clinically relevant resistance factor and highlight its inhibition as a promising strategy to overcome cisplatin resistance in NSCLC.

FDA-approved Topo IIα inhibitor etoposide exerts anticancer activity by stabilizing Topo IIα-mediated temporary chromosome bridge breaks, ultimately inducing cytotoxic DSB [100,157]. It is widely used in standard regimens for multiple malignancies such as small-cell lung cancer (SCLC) and ovarian germ cell tumor [158,159]. Some cancer cells depend on upregulated Topo IIα for its elevated activity to sustain their aggressive proliferation [160]. Indeed, etoposide exhibits markedly greater efficacy in tumors with high Topo IIα activity compared with those expressing low levels. This was validated by the restoration of etoposide sensitivity by Topo IIα overexpression in the etoposide-resistant human brain tumor cells [161,162,163,164]. SMURF2 functions as a key upstream regulator that maintains Topo IIα abundance, thereby promoting the Topo IIα-mediated chromosomal breakage events required for robust etoposide-induced cell death. Therefore, SMURF2-mediated stabilization of Topo IIα could likewise enhance etoposide responsiveness in tumors.

**Figure 5:**
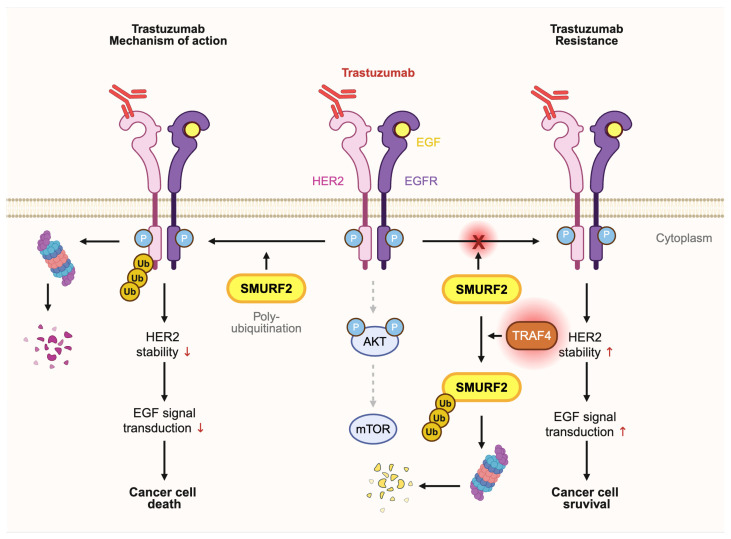
SMURF2-dependent regulation of HER2 stability determines trastuzumab sensitivity. Under normal conditions (**left**), SMURF2 promotes HER2 polyubiquitination and degradation, thereby enhancing trastuzumab-mediated suppression of AKT/mTOR signaling. This results in reduced HER2 stability, decreased cell proliferation, and increased apoptosis. When TRAF4 is overexpressed (**right**), it ubiquitinates and degrades SMURF2, preventing HER2 polyubiquitination and allowing HER2 to accumulate despite trastuzumab treatment. Sustained HER2 signaling maintains AKT/mTOR activity, driving continued proliferation and reduced apoptosis, thereby promoting trastuzumab resistance (created with Biorender.com, accessed on 6 January 2026).

The polyubiquitination of human epidermal growth factor receptor 2 (HER2) mediated by SMURF2 can increase trastuzumab sensitivity in HER2-positive breast cancer. Trastuzumab is a monoclonal antibody that binds to the extracellular domain of HER2 and suppresses signal transduction pathways, such as the AKT/mTOR pathway, to induce cell proliferation arrest and apoptosis (Figure 5) [165,166,167]. As one of the most widely used and clinically validated targeted therapies in oncology, trastuzumab has dramatically improved survival outcomes in HER2-positive breast cancer and remains the standard-of-care backbone for both early-stage and metastatic disease. However, one of the mechanisms that breast cancer cells maintain the HER2 signaling even with the trastuzumab treatment is the overexpression of TRAF4 that can downregulate SMURF2 expression by UPS [111,168,169]. TRAF4-mediated ubiquitination of SMURF2 promotes its proteasomal degradation, and the resultant loss of SMURF2 prevents HER2 from undergoing SMURF2-dependent polyubiquitination and degradation, thereby allowing HER2 to accumulate and sustain downstream oncogenic signaling even in the presence of trastuzumab (Figure 5). These observations further suggest that SMURF2 expression could serve as a predictive biomarker, identifying tumors in which TRAF4 inhibition by restoring SMURF2 stability may synergize with trastuzumab to overcome resistance and enhance therapeutic responsiveness [169].

SMURF2 is a major factor that contributes to Erlotinib resistance, an EGFR tyrosine kinase inhibitor (TKI) [137,138]. Erlotinib is a first-generation EGFR TKI, and it can bind and inhibit the uncontrolled EGFR signal transduction by suppressing the autophosphorylation of the tyrosine kinase domain of EGFR with the L858R mutation [139]. Clinically, it has served as a foundational targeted therapy for NSCLC with L858R or exon 19 deletion mutations, establishing EGFR-targeted therapy as a standard treatment approach in lung cancer. SMURF2 can stabilize EGFR, including the TKI-sensitive L858R and the TKI-resistant T790M variants, through K63-linked polyubiquitination, preventing its acetylation-dependent internalization and subsequent lysosomal degradation [137,138,140,141]. This stabilization preserves EGFR surface expression and maintains downstream signaling activity even in the presence of EGFR TKIs, thereby promoting therapeutic resistance. Knockdown of SMURF2 restores erlotinib sensitivity in erlotinib-resistant non-small cell lung cancer cells by disrupting the SMURF2-mediated polyubiquitination that normally stabilizes mutant EGFR [138]. When SMURF2 levels are reduced, EGFR is no longer protected from internalization and instead undergoes endocytosis followed by endosomal sorting and lysosomal degradation, leading to a marked decrease in functional EGFR molecules at the cell surface [137,142]. As a result, diminished EGFR signaling allows erlotinib to effectively inhibit the remaining receptor population, thereby restoring drug responsiveness.

SMURF2 is an upstream regulator that promotes the emergence of Mitogen-activated protein kinase kinase (MEK) inhibitor resistance in melanoma [123]. MEK inhibitors, such as selumetinib, suppress RAS–MEK–ERK/MAPK-driven proliferation by blocking ERK1/2 phosphorylation, which reduces cyclin D1–CDK4/6 signaling, leading to G1 arrest and cytostatic or cytotoxic effects [124,125]. Selumetinib is FDA-approved for NF1-associated plexiform neurofibromas and occasionally used in RAS/BRAF-driven tumors. However, some melanoma cells develop resistance by increasing the expression of Paired Box 3 (PAX3) and its transcriptional target Microphthalmia-associated transcription factor (MITF), which promotes cell cycle progression and suppresses apoptosis [125,126,127,128,129]. Higher *SMURF2* mRNA and protein levels have been observed in MEK inhibitor-resistant melanoma cell lines, and *PAX3* mRNA level is positively correlated with SMURF2 expression level [125]. SMURF2 is involved in resistance by inhibiting the SMAD2/3 transcription factor complex that downregulates *PAX3* and *MITF* [125,130]. The combination of SMURF2 deletion and MEK inhibitors more sensitized melanoma cells and enhanced the cytotoxic effect of MEK inhibitors on melanoma cells [125].

## 5. SMURF2-Related Novel Directions of Cancer Theranostics

### 5.1. Inhibition of Upregulated SMURF2

SMURF2 is promising as a biomarker and therapeutic target for cancer diagnosis and therapy because cancer development and prognosis have been found to be significantly correlated with its expression level in some cancers. Direct or indirect regulation of SMURF2 may be a promising strategy for precision medicine. Using lung cancer cell lines, Chae et al. have shown that miR-195 and miR-497 target SMURF2 at the 3′-untranslated region (UTR) and inhibit its expression, and increase TβRI stability as well as its target gene, CDKN1A (p21) [144]. Tumor suppressive miR-195 and miR-497 were downregulated in NSCLC, and the authors showed a potential therapeutic strategy by elucidating the direct connection to SMURF2 and the TGF-β signaling. Although microRNA-based therapeutics are still not widely applied in clinical settings due to delivery and safety challenges, they provide a potential therapeutic strategy for high SMURF2-expressing or downregulated TGF-β-p21 cancer types [170,171,172,173]. These findings support the feasibility of using viral miRNA-replacement approaches to downregulate SMURF2 in tumors with SMURF2 overexpression.

Curcumin, a well-established natural polyphenolic compound, has recently been reported to exert anticancer effects through suppression of SMURF2 activity. Xi et al. demonstrated, using lung cancer cell lines, the effect of curcumin on SMURF2, resulting in pronounced anticancer effects both in vitro and in vivo. Notably, curcumin administration led to a marked reduction in tumor volume and tumor weight in NSCLC xenograft models, accompanied by decreased proliferative activity [174]. Molecular docking simulations have reported that curcumin interacts with the HECT domain of SMURF2, the catalytic region responsible for its E3 ubiquitin ligase activity, providing a structural basis for SMURF2 inhibition [174]. These findings highlight that curcumin may exert therapeutic benefit by modulating SMURF2 activity, particularly in tumors characterized by SMURF2 upregulation.

Small-molecule inhibitors of SMURF2 have also been reported. Protein-based inhibitory strategies include the design of ubiquitin variants (UbVs) such as S2.4, which is a synthetic variant of ubiquitin engineered to bind specifically to SMURF2 with high affinity. In HEK293 cells, it exhibited inhibitory activity toward SMURF2 by blocking the E2–E3 interaction, thereby abolishing its ubiquitin ligase function. Structural analyses revealed that UbV S2.4 directly binds to the E2-binding region of SMURF2, leading to functional inhibition [175]. These findings suggest the potential of protein-based inhibitors such as UbV S2.4 as therapeutic candidates. In parallel, small-molecule inhibitors such as Heclin, a HECT ligase inhibitor, provide one of the first clear demonstrations that HECT ligases can be pharmacologically targetable. It suppressed the catalytic activity of multiple HECT ligases, including SMURF2, and was cytotoxic to HEK293 cells [176,177]. It induces conformational changes that oxidize the catalytic cysteine residue within the HECT domain, thereby disrupting enzymatic activity. Such small-molecule inhibitors further highlight SMURF2 as a pharmacological target for cancer therapy.

### 5.2. Pathways Enhancing Downregulated SMURF2

Unlike inhibiting SMURF2 overexpression, which can be achieved through direct inhibition strategies, therapeutic upregulation of SMURF2 is more challenging. When the tumor suppressive SMURF2 is downregulated, upstream regulatory pathways are worth investigating. Although still in the investigative stage, the studies discussed in this section may provide a future direction for targeting SMURF2-related pathways for cancer therapy.

In smoking-induced pancreatic cancer cells, cigarette smoke extract (CSE) upregulates YBX1 expression, which promotes the expression of CBX3 that suppresses SMURF2 expression, resulting in pancreatic cancer progression [120]. These findings indicate that smoking-related pancreatic cancers characterized by YBX1/CBX3 upregulation and low expression of SMURF2 may represent a distinct patient subset in which restoring SMURF2 expression could be therapeutically beneficial. This expression pattern may also serve as a companion biomarker for patient selection.

In colorectal cancer (CRC), SMURF2 expression level was analyzed in a specific group of 66 CRC patients who had surgery for both primary CRC and liver metastasis. As a result, low SMURF2 expression was observed in more than half of the primary tumors in the cohort, and in some metastasized tumors in the cohort [73]. Patients with downregulated SMRUF2 expression showed shorter overall survival and disease-free survival time, suggesting that SMURF2 has the potential to be a therapeutic target. Moreover, SMURF2 expression level may be a promising biomarker for the diagnosis and prognosis of CRC. There are some potential strategies that can be suggested for CRC treatment. SMURF2 downregulates carbohydrate response element-binding protein (ChREBP), which is a glucose-responsive transcription factor that promotes cell proliferation by upregulating the aerobic glycolysis pathway [113,114]. The aerobic glycolysis pathway is the major energy source of cancer [115]. Li et al. demonstrated that the suppression of aerobic glycolysis by SMURF2-mediated ChREBP inhibition suppressed tumor growth, and AKT was identified to be an upstream negative regulator of SMURF2 [114]. Since direct upregulation of SMURF2 is challenging, this suggests that an AKT inhibitor could be a potential CRC therapy strategy for those who have low SMURF2 and high ChREBP expression by indirectly restoring SMURF2 levels.

SMURF2 can directly interact with Ras homology family member A (RhoA) and indirectly with angio-associated migratory cell protein (AAMP), which contribute to the metastatic properties of CRC cells [116,117]. SMURF2 mediates ubiquitination and degradation of RhoA, resulting in decreased migration of the CRC cells, but AAMP competitively binds to RhoA and inhibits these functions [117]. In this context, reducing the AAMP expression level or disrupting the interaction of AAMP and RhoA is a possible strategy for metastatic CRC, as targeting this interaction may help suppress migratory and invasive cancer behavior.

SMURF2 and ubiquitin-specific peptidase 47 (USP47), a deubiquitinating enzyme, competitively bind and regulate special AT-rich sequence-binding protein-1 (SATB1) stability [118]. SATB1 promotes the transcription of genes involved in colon cancer development and metastasis, whereas SMURF2 decreases SATB1 stability through ubiquitin-mediated degradation, thereby reducing tumor cell growth and suppressing metastatic potential [118,119]. In contrast, USP47 removes ubiquitin chains from SATB1 and stabilizes it, leading to enhanced SATB1-driven oncogenic transcriptional programs [118]. In colon cancer cell lines, *USP47* knockdown was shown to reduce cell proliferation, while SMURF2 overexpression suppressed migration and invasion; importantly, these phenotypic effects were largely mediated by SATB1 downregulation. This reciprocal regulation suggests that therapeutic strategies aimed at inhibiting USP47 or boosting SMURF2 activity may effectively provide anticancer benefit in SATB1-dependent colon tumors.

These highlighted studies are mainly cell-based and in the basic research stage but are worth continuing investigation. Like the case of p53 and MDM2, inhibiting the negative regulator of the target protein to enhance its function is a strategy that can be applicable to low SMURF2-expressing cancers. Although still in the investigative stage, these studies may provide future strategies for SMURF2 targeted therapy.

## 6. Summary

SMURF2 is an E3 ubiquitin ligase that is expressed at different levels and plays a role as a tumor-promoting or tumor-suppressive factor depending on the specific cancer type and cellular context. For this reason, deep analysis and understanding of the complicated characteristics of SMURF2 and its related cellular pathways are essential to design effective and innovative strategies for cancer diagnosis and treatment.

SMURF2 expression level is a promising biomarker for diagnosis and prognosis because it has critical roles in multiple homeostatic networks, including TGF-β signaling, Hedgehog regulation, DNA damage response, chromatin maintenance, and cell-cycle control, whcih are directly or indirectly linked to cell survival, proliferation, and migration. Dysregulated SMURF2 expression, whether reduced or overexpressed, reprograms these regulatory circuits toward phenotypes such as aberrant proliferation, EMT, genomic instability, and therapy resistance.

The oncogenic or tumor suppressive role of SMURF2 is somewhat complex in its involvement in the TGF-β signaling pathway. In most epithelial cells, the TGF-β signaling leads to cell cycle regulation and apoptosis, and during development, although it is involved in cell proliferation, it also drives differentiation. These functions are mostly tumor suppressive in nature, and overexpression of SMURF2 in this axis would be oncogenic, one example being downregulation of CDKN1A. On the other hand, in neoplastic cells where stem cell-like properties are re-emerging, downregulation of SMURF2 would be oncogenic, as the TGF-β pathway expresses genes such as MYC, which are upregulated in stem cells. In addition, low levels of SMURF2 can over-stabilize ID proteins, which is a major factor in maintaining multipotency of stem cells, and is often upregulated in many cancers [58]. As discussed earlier, increased levels of ID2 can lead to decreased p21, resulting in dysregulation in cell cycle control. Cancer stem cells are often related to poor prognosis, as they allow cancer cells to continue to evolve and proliferate and induce drug resistance. ID is a transcription factor, hard to target. However, with the SMRUF2 regulation discussed in this paper, we can develop a strategy to target ID upregulated cells. For invasive and metastatic cancers, restoring SMURF2, possibly by targeting its upstream regulators such as TRAF4, could be a possibility. Although still in the investigative stage, potential therapeutic strategies targeting the upstream regulation of SMURF2 would be worth pursuing.

The Hedgehog signaling pathway is essential for embryonic development but is minimally active in adult cells [105,178]. Its activators are considered proto-oncogenes, and SMURF2 restricts overactivation of the Hedgehog signaling by destabilizing RNF220, repressing transcription of GLI1 target genes. Downregulation of SMURF2 in this axis is oncogenic, as the Hedgehog signaling promotes cell growth. Other than its transcriptional regulation, SMURF2 is involved in genome maintenance by stabilizing Topo IIα. The monoubiquitination and stabilization of the genome make SMURF2 tumor suppressive in this context. Although its downregulation may increase genome instability, this can be taken advantage of in cisplatin resistance cases to increase drug sensitivity.

The development of resistance to cancer in existing cancer treatments is a big problem that must be overcome. Since SMURF2 has been studied to be involved in the molecular pathways of some cancer treatments, targeting SMURF2 has the possibility to restore and improve cancer treatment effectiveness and sensitivity. For instance, SMURF2 restrains miR-15b–TGF-β-driven EMT and preserves gemcitabine sensitivity, yet its stabilization of Topo IIα or mutant EGFR contributes to resistance against cisplatin and EGFR TKIs, respectively. Similarly, SMURF2 enhances responsiveness to etoposide by maintaining Topo IIα. Thus, SMURF2-targeted interventions will require context-specific approaches. In addition to SMURF2 expression level, it would be especially important to evaluate target genes of the TGF-β signaling pathway, ID target genes, specifically analyzing whether stem cell and EMT markers are elevated, or cell cycle regulators such as *CDKN1A* are downregulated. Additionally, expression levels of SMURF2-related proteins, such as Topo IIα, HER2 should be co-analyzed to enhance drug sensitivity. These analyses would elucidate the most effective strategy to target SMURF2-related pathways for the specific cancer type. Such precision strategies hold strong potential for improving diagnosis, overcoming therapeutic resistance, and enabling effective SMURF2-guided cancer treatment.

## Figures and Tables

**Table 1:** Context-dependent SMURF2 dual role in carcinogenesis.

Cellular Pathways	SMURF2-Dependent Key Mechanism	Cellular Context (Cancer Type)	SMURF2 Expression Alteration	Oncogenic Mechanism	Impact on Carcinogenesis	Reference
CRC proliferation & metastasis- related pathways	Polyubiquitination of ChREBP, RhoA, and SATB1 for proteolysis	Colorectal cancer	Downregulation	Loss of SMURF2 ability to suppress ChREBP, RhoA, and SATB1	Upregulation of aerobic glycolysis pathway, and enhanced metastasis	[73,113,114,115,116,117,118,119]
TGF-β signaling	Polyubiquitination of TGF-β receptor for proteolysis	Lung cancer	Upregulation	Downregulated miR-195/-497, leading to elevated SMURF2 expression (increased TGF-β receptor degradation)	Suppressed TGF-β– induced p21 expression	[75,86,87]
		Glioblastoma	Downregulation	Impaired SMURF2 ability to degrade the TGF-β receptor	Increased stemness, invasiveness, and tumorigenicity of GSCs	[80,109]
		Pancreatic cancer (smoking-related)	Downregulation	Suppression of SMURF2 transcription by CBX3, leading to excessive activation of TGF-β signaling	Tumor-promoting TGF-β signaling and cancer progression	[120,121,122]
	Transcription regulation by mono-ubiquitination of SMAD2/3	Melanoma (MEK- inhibitor resistant)	Upregulation	Upregulated PAX3-MITF pathway due to the dysregulation of TGF-β signaling	Promoted cell cycle progression, and the suppression of apoptosis	[123,124,125,126,127,128,129,130]
Protein relocalization and stability regulation	Tight regulation of its cellular localization and related-mitogenic pathways	Breast cancer, Prostate cancer	Upregulation	Increased SMURF2 stability and relocalization in cytoplasm	Activation of growth-promoting mitogenic pathways	[83]
Genome stability-related pathways	Polyubiquitination of RNF20 for proteolysis	Breast cancer, lymphoma	Downregulation	Excessive RNF20-driven H2Bub1	Overly decondensed chromatin and impaired DSB repair	[76,103]
	Stabilization of Topo IIα	Breast, colorectal, hepatocellular cancer	Downregulation	Decreased Topo IIα protein stability	Defective decatenation, persistent chromosomal bridge formation, and increased genomic instability	[100,101,102]
ID2-E2A-p21 pathway	Polyubiquitination of ID2 for proteolysis	Lung cancer	Downregulation	Dysregulation of ID2-E2A-p21 pathway	Diminished p21 expression, leading to loss of G1 cell cycle restraint and sustained proliferation	[97]
Hedgehog signaling	Polyubiquitination of RNF220 for proteolysis (indirectly enhances PRC2 activity)	Sonic hedgehog-type medulloblastoma	Downregulation	Dysregulation of RNF220-PRC2-GliA signaling pathway	Shift of neural progenitor maintenance to malignant growth	[104,105,106,131]
Growth factor signaling	Mono-ubiquitination of UBCH5 for the complex formation and polyubiquitination of β-TrCP1 for proteolysis	Lung cancer, colorectal cancer	Upregulation	KRAS-oncogenic pathway activation by SMURF2-UBCH5-β-TrCP1-KRAS axis	Promoted cell proliferation	[132,133,134,135,136]
	Polyubiquitination of EGFR for the stabilization of its cell surface expression	Non-small-cell-lung cancer (TKI- resistant)	Upregulation	Mutant EGFR stabilization by SMURF2	Sustained EGFR surface expression and signaling	[137,138,139,140,141,142]

## Data Availability

No new data were created or analyzed in this study. Data sharing is not applicable to this article.

## Abbreviations

The following abbreviations are used in this manuscript:

SMURF2	Smad ubiquitin regulatory factor 2
TNBC	Triple-negative breast cancer
T-ALL	T-cell acute lymphoblastic leukemia
HNSCC	head and neck squamous cell carcinoma
RING	Really Interesting New Gene
CDKIs	Cyclin-Dependent Kinase Inhibitors
GSCs	Glioma stem cells
CIN	chromosomal instability
TβR1	TGF-β type I receptor
EMT	epithelial-mesenchymal transition
NSCLC	non-small-cell lung cancer
SCLC	small-cell lung cancer
HER2	human epidermal growth factor receptor 2
EGFR TKI	epidermal growth factor tyrosine kinase inhibitor
MEK	Mitogen-activated protein kinase
ccRCC	clear cell renal cell carcinoma
CNKSR2	connector enhancer of kinase suppressor of Ras 2
UbVs	ubiquitin variants
ChREBP	carbohydrate response element-binding protein
AAMP	angio-associated migratory cell protein
USP47	ubiquitin-specific peptidase 47
